# Are the Stage and the Incidental Finding of Endometriosis Associated with Fallopian Tube Occlusion? A Retrospective Cohort Study on Laparoscopic Chromopertubation in Infertile Women

**DOI:** 10.3390/jcm11133750

**Published:** 2022-06-28

**Authors:** Daniel Mayrhofer, John Preston Parry, Marlene Hager, Klara Beitl, Christine Kurz, Johannes Ott

**Affiliations:** 1 Clinical Division of Gynecologic Endocrinology and Reproductive Medicine, Medical University of Vienna, 1090 Vienna, Austria; daniel.mayrhofer@meduniwien.ac.at (D.M.); marlene.hager@meduniwien.ac.at (M.H.); klara.beitl@meduniwien.ac.at (K.B.); christine.kurz@meduniwien.ac.at (C.K.); 2 Parryscope and Positive Steps Fertility, Madison, MS 39110, USA; drprestonparry@gmail.com; 3 Department of Obstetrics and Gynecology, University of Mississippi Medical Center, Jackson, MS 39216, USA

**Keywords:** chromopertubation, endometriosis, fallopian tube, fertility

## Abstract

Endometriosis seems to have a strong negative effect on female fertility. The aim of this study was to assess the rate of tubal occlusion diagnosed via laparoscopic chromopertubation in infertile women with endometriosis and compare the results to infertile women without endometriosis. In this retrospective cohort study, 275 infertile women with endometriosis and 49 infertile women without endometriosis undergoing diagnostic laparoscopy for primary or secondary infertility with chromopertubation at the Medical University of Vienna between January 2012 and December 2020 have been investigated. During the laparoscopic assessment of tubal patency, significantly more fallopian tubes were occluded in the endometriosis group compared to the control group (25.8 versus 15.3%; *p* = 0.029). Unilateral and bilateral occlusion was found significantly more often in patients with endometriosis (*p* = 0.021). In the multivariate analysis, only the rASRM stage (the revised classification of endometriosis by the American Society for Reproductive Medicine) showed a significant association with bilateral occlusion (OR 1.400, 95%CI: 1.018–1.926; *p* = 0.038). Both a higher rASRM stage (OR 2.181, 95%CI: 1.191–3.995; *p* = 0.012) and secondary infertility (OR 1.514, 95%CI: 1.156–1.983; *p* = 0.003) were associated with an increased risk for any kind of fallopian tube occlusion. Endometriosis seems to be associated with an increased risk for fallopian tube occlusion. The rate of tubal occlusion increased significantly with the rASRM stage.

## 1. Introduction

The overall prevalence of endometriosis in population-based studies varies from 0.8 to 6% [1,2,3]. In contrast, a considerably higher prevalence has been reported for subfertile women with ranges from 20 to 50% [4,5], strongly consistent with endometriosis having a negative effect on female subfertility and infertility. As recently reviewed [6], several factors are thought to contribute to the decreased fertility in women with endometriosis. These include, but are not limited to, chronic intraperitoneal inflammation which may lower follicular quality and quantity, suboptimal sperm motility, damage to sperm DNA, interference with oocyte–sperm binding, impaired embryo development and implantation, dysfunction of the hypothalamic–pituitary–ovarian axis, reduced expression of progesterone receptors in the endometrium which may cause progesterone resistance, and dysfunction of tubal peristalsis [6], or even an increased risk for fallopian tube blockage [7,8]. While oocyte donation studies suggest that endometrial receptivity and the capacity for implantation seem minimally or unaltered in women with endometriosis [9], these other factors likely contribute to subfertility of affected patients.

However, whether endometriosis in general is associated with fallopian tube occlusion remains controversial. In a cohort study, 7/20 (35.0%) women with moderate or severe endometriosis revealed tubal obstruction in contrast to 22/124 (17.7%) women without this condition. Though this was a doubling in the rate of occlusion, this difference did not reach statistical significance (*p* = 0.07) [10]. Another retrospective study reported that both endometriosis and retrograde menstruation were linked to tubal damage [11]. A recent larger study revealed that women with endometriosis of any severity had higher rates of both unilateral and bilateral tubal occlusion. Moreover, endometriosis affecting a fallopian tube is associated with a high rate of ipsilateral tubal occlusion and moderate endometriosis (the revised score of the American Society of Reproductive Medicine, rASRM, III) is associated with higher risk for tubal occlusion relative to rASRM stages I and II [8]. Additionally, in a recent retrospective cohort of women with polycystic ovary syndrome who underwent laparoscopic chromopertubation, incidental endometriosis was associated with tubal occlusion, despite most patients having low-stage disease [11]. 

In addition to the uncertainty as to whether endometriosis would really be a causative factor for tubal blockage, the role of incidental endometriosis, i.e., endometriosis without pain symptoms, for female fertility remains unclear. The prevalence of incidental endometriosis has been reported to range from 7.7 to 45.3% [11,12,13]. One important study suggested that incidental endometriosis is relevant, as 50% of patients with recurrent implantation failure after IVF for unexplained infertility and incidental endometriosis subsequently conceived naturally after complete resection [14]. Nevertheless, the literature on incidental endometriosis is scarce and its effect on tubal function is unknown.

To address these limitations in the literature, we evaluated the rate of tubal occlusion as diagnosed by laparoscopic chromopertubation in infertile women with endometriosis, with a special focus on the rASRM stage and incidental endometriosis. In addition, we compared these results to a control group of women who also suffered from infertility but underwent laparoscopy for the treatment of a follicular ovarian cyst only.

## 2. Materials and Methods

### 2.1. Patient Population

In a retrospective cohort study, all women, aged 18–45 years undergoing diagnostic laparoscopy for primary or secondary infertility at the Clinical Division of Gynecologic Endocrinology and Reproductive Medicine of the Medical University of Vienna, Austria, from January 2012 to December 2020, were included. All women with laparoscopically and histologically confirmed endometriosis of any stage (*n* = 275) were eligible for inclusion. For the control group, all women undergoing the same surgical procedure without endometriosis but with at least one laparoscopically and histologically confirmed follicular ovarian cyst were included (*n* = 49). Inclusion and exclusion criteria for both groups are shown in Table 1. The study was approved by the Institutional Review Board of the Medical University of Vienna (IRB number 2319/2020). 

### 2.2. Surgical Technique

All surgical procedures were conducted under general anesthesia as reported previously and were either directly performed or supervised by experts in infertility surgery [15,16]. Before laparoscopy, a Spackmann uterine manipulator with clamp fixation and a rubber cone with 18 mm diameter (Reference number 1264; WISAP^®^ Medical Technology GmbH, Brunnthal/Hofolding, Germany) was placed through the cervix and advanced to 10 mm from the uterine fundus for each patient. After establishing a pneumoperitoneum (intraabdominal pressure: 12 mmHg) and a thorough examination of the liver region, pelvis, and internal genitalia, chromopertubation was performed by injecting dilute solution of indigo carmine blue dye (Amino AG, Gebenstorf, Switzerland) through the uterine manipulator using a 50 mL syringe. Tubal patency was assessed by the passage of blue dye through the fimbrial ends of each fallopian tube. Parameters recorded included tubal patency and the subjective pressure needed for chromopertubation.

### 2.3. Parameters Analyzed

The main outcome parameter was fallopian tube patency assessed by laparoscopic chromopertubation and documented independently for both sides. When tubal occlusion was identified, the localization was documented and classified as either proximal or distal occlusion, as previously reported [15]. Additional information collected included the presence of “incidental endometriosis”, defined as endometriosis without pelvic pain (i.e., dysmenorrhea NRS ≤ 3, dyspareunia NRS ≤ 3) and without sonographic signs of endometriosis, with particular emphasis on endometriomas but also accounting for deep infiltrating endometriosis. Endometriosis was classified according to the revised American Society of Reproductive Medicine score (rASRM) [17]. Moreover, information on patients’ age, body mass index (BMI), type of infertility (primary versus secondary), presence of peritubal adhesions which led to tubal distortion, and subjective assessment of pressure needed to achieve fallopian tube patency (low or high) has also been collected. 

### 2.4. Statistical Analysis

Variables were described by numbers (frequencies) and mean ± standard deviation. Statistical analysis was performed with SPSS 28.0 for Windows (IBM Corp., Armonk, NY, USA, 1989–2022) using the Fisher’s exact test or the Chi-square for categorical parameters. Univariate binary logistic regression models were used to test the predictive value of all coefficients for the presence of fallopian tube occlusion. Significant parameters were entered in a multivariate logistic regression model. Odds ratios (OR) and their 95% confidence intervals (95%CI) are given. Differences were considered statistically significant if *p* < 0.05.

## 3. Results

No differences between women in the endometriosis and the control groups were observed for the mean patient age at the time of surgery (32.2 ± 5.2 versus 32.5 ± 5.0 years, respectively; *p* = 0.730), BMI (23.9 ± 4.4 versus 23.4 ± 5.0 kg/m^2^, respectively; *p* = 0.627), and type of infertility (secondary infertility 59/275, 21.5% versus 9/49, 18.4%, respectively; *p* = 0.707). 

For the endometriosis group only, 29.8% (82/275) of patients reported no pelvic pain, while 14.9% (41/275) cases were considered “incidental”. According to the rASRM, stages I, II, III, and IV were found in 97 (35.5%), 63 (22.9%), 90 (32.7%), and 25 (9.1%) patients, respectively (Table 2). Notably, 132/275 women (48.0%) had at least one endometrioma, and in 29/275 women (10.5%), deep infiltrating endometriosis was found. In 13 women with endometriosis (4.7%), uni- or bilateral peritubal adhesions, which distorted the affected tube(s), were found. There were no distended fallopian tubes.

With laparoscopic chromopertubation, 142/550 (25.8%) tubes were occluded in the endometriosis group compared to 15/98 (15.3%) in the control group (*p* = 0.029). There were no differences in the rates of women with unilateral or bilateral tubal occlusion (*p >* 0.05). However, any kind of tubal occlusion (either unilateral or bilateral) was found more often in endometriosis patients (35.3%) than in controls (20.4%; *p* = 0.021), as described in Table 3.

For the multivariate analysis, several parameters possibly associated with fallopian tube occlusion were evaluated in endometriosis patients. For bilateral occlusion, only the rASRM stage was significant (OR 1.400, 95%CI: 1.018–1.926; *p* = 0.038; Table 4). However, for the presence of any kind of fallopian tube occlusion, i.e., either unilateral or bilateral, both a higher rASRM stage (OR 2.181, 95%CI: 1.191–3.995; *p* = 0.012) and secondary infertility (OR 1.514, 95%CI: 1.156–1.983; *p* = 0.003) were associated with increased risk in the multivariate analysis (Table 5). Notably, endometriosis being an incidental finding did not alter the risk for fallopian tubal occlusion (Table 4 and Table 5). Also notably, the rate of women with both unilateral and bilateral fallopian tubal occlusion increased significantly with the rASRM stage, as shown in Table 6. 

## 4. Discussion

In this retrospective cohort study, sterile women with endometriosis demonstrated a high rate of fallopian tube occlusion, where over one third of these patients (35.3%) had at least one closed tube. This was significantly higher than in infertile control patients presenting for follicular ovarian cysts (20.4%; Table 3). Moreover, the rate of bilaterally occluded tubes was significantly higher in endometriosis patients (25.8 versus 15.3% of all tubes). As mentioned above, the literature about endometriosis and the risk for tubal occlusion is diverse [10,11]. Possible pathophysiological explanations describe negative effects caused by the mechanical effects of endometriosis, leading to a distorted micro-anatomy of the fallopian tubes. Intraabdominal adhesions and dysperistalsis of the tubal myometrium may also hinder the tubo-ovarian contact and alter uterotubal transport [6,18]. Notably, our data are in line with those of a recent study, which demonstrated tubal obstruction rates of 35.0% in women with moderate or severe endometriosis and 17.7% in women without endometriosis [10]. 

In addition, one major finding of our data is that a higher rASRM stage was associated with an increased risk for tubal occlusion (Table 4, Table 5 and Table 6). This is quite similar to another recent report by Nicolaus et al. where rASRM stage III was associated with the highest risk for tubal occlusion, occurring in 38.2% on the right side and in 47.1% on the left [8]. At least according to the data set newly presented in our submission, the endometriosis stage seems to be the most important predictor for the risk of fallopian tube occlusion (Table 4 and Table 5). It seems worth mentioning that about 50% of women with moderate and severe endometriosis revealed at least unilateral fallopian tube occlusion (Table 6). Though IVF is often recommended for infertile women with endometriosis, especially when stages III or IV are found, understanding tubal status has meaningful implications for efficacy should patients choose different methods for conception. Additionally, women with minimal and mild endometriosis revealed high rates of tubal occlusion. In detail, about one fourth of these patients had at least one occluded tube and about 12% suffered from bilateral occlusion (Table 6). Although these rates are comparable to those found in the control group (Table 3), even the finding of a unilateral occlusion might be considered more clinically relevant when endometriosis is also present in a patient and physicians might offer IVF earlier when several causes of subfertility are found. Moreover, perspective regarding the multifactorial effects of endometriosis on fertility are essential to informed autonomy.

Apart from the rASRM stage, only secondary infertility was a significant factor for the presence of any kind of fallopian tube occlusion (Table 5). Some studies have shown that women with secondary infertility have a higher likelihood of having tubal occlusion, at least on hysterosalpingography, compared to those with primary infertility, with an adjusted risk ratio of 1.75 [19]. These findings are more often related to surgical causes than in woman with primary infertility, who, in contrast, show higher rates of congenital causes of tubal occlusion [20,21,22].

Notably, another tested parameter was whether the laparoscopic finding of endometriosis was incidental, i.e., without any self-reported associated pain symptoms and sonographic suspicion of the disease. As recently demonstrated, incidental endometriosis was found to increase the risk for fallopian tube occlusion in women with polycystic ovary syndrome compared to non-endometriosis patients [11]. However, according to the uni- and multivariate binary logistic regression models presented in Table 4 and Table 5, incidental endometriosis did not lower the risk for tubal occlusion compared to symptomatic endometriosis, which had already been suspected before laparoscopy. 

Several study limitations should be acknowledged. First, the sample size in the control group should be mentioned. However, it is hard to find a completely valid control group for laparoscopic chromopertubation, especially in this setting. All women in the endometriosis group were infertile; thus, the subjects in the control group also need to be infertile. One of the indications for laparoscopy least likely to affect fallopian tube integrity would be benign ovarian cysts. Though commonly used, otherwise unexplained infertility is arguably an unsuitable control population, as unexplained infertility often relates to tubal disease that has been missed with conventional testing [23]. Similarly, polycystic ovary syndrome patients are at greater risk for altered tubal function and are in need of higher chromopertubation pressure to achieve patency [24]. Additionally, the retrospective study design must be considered a limitation. The lack of data on current or past sexually transmitted infections or pelvic inflammatory disease must also be considered a limitation, as these factors might also be a cause for tubal occlusion. Finally, one could consider it a study limitation that other possible causes of infertility were ruled out by applying them as exclusion criteria. Due to these specific characteristics of the study group, our data only apply for endometriosis patients with concurrent infertility unless confirmed for non-infertile women in the future. In a potential forthcoming prospective study, a more suitable control group as well as enrollment of endometriosis patients without infertility should help with exploring the role of fallopian tube blockage in endometriosis patients in further detail.

## 5. Conclusions

In conclusion, in infertile women, endometriosis seems to be associated with an increased risk for fallopian tube occlusion. While the prevalence of bilateral tubal occlusion is surprisingly high in patients with minimal or mild endometriosis, it appears to be significantly higher in women with moderate and severe endometriosis. 

## Figures and Tables

**Table 1:** Inclusion and exclusion criteria of study population and control group.

	Endometriosis Group	Control Group
**Inclusion criteria**	Women aged 18–45 years Underwent diagnostic laparoscopy for primary or secondary infertility January 2012–December 2020 Endometriosis of any grade was found (confirmed laparoscopically and histologically)	Women aged 18–45 years Underwent diagnostic laparoscopy for primary or secondary infertility January 2012–December 2020 Follicular ovarian cyst (confirmed laparoscopically and histologically)
**Exclusion criteria**	PCOS (polycystic ovary syndrome) One or both tubes missing before the operation Hydrosalpinx of one or both tubes Myomas, uterine malformation (because these could hypothetically have an impact on tubal function) Couples with male factor (abnormal sperm test; in order to increase the chance of endometriosis being the major factor of the underlying infertility)	PCOS (polycystic ovary syndrome) One or both tubes missing before the operation Hydrosalpinx of one or both tubes Myomas, uterine malformation (because these could hypothetically have an impact on tubal function) Couples with male factor (abnormal sperm test) Dermoid cysts Note: Endometriosis of any grade was excluded laparoscopically

**Table 2:** Distribution of basic patient characteristics.

	Endometriosis Patients (*n* = 275)	Controls (*n* = 49)	*p*
Mean age at surgery (years)	32.2 ± 5.2	32.5 ± 5.0	0.730
Body mass index (kg/m^2^)	23.9 ± 4.4	23.4 ± 5.0	0.627
Type of infertility (secondary infertility)	59 (21.5%)	9 (18.4%)	0.707
	**Endometriosis patients (*n* = 275)**		
No pelvic pain	82 (29.8%)		
“Incidental” endometriosis	41 (14.9%)		
rASRM (stages)			
I	97 (35.5%)		
II	63 (22.9%)		
III	90 (32.7%)		
IV	25 (9.1%)		

**Table 3:** Results of laparoscopic chromopertubation in women with endometriosis and controls.

	Endometriosis Patients (*n* = 275)	Controls (*n* = 49)	*p*
Unilateral tubal occlusion	52 (18.9)	5 (10.2)	0.159
Bilateral tubal occlusion	45 (16.4)	5 (10.2)	0.297
One or both tubes closed	97 (35.3)	10 (20.4)	*0.021*

**Table 4:** Prediction of bilateral fallopian tube occlusion in women with endometriosis using a binary logistic regression model.

	Bilateral Occlusion	One or Both Tubes Open	Univariate Analysis			Multivariate Analysis		
	(*n* = 45)	(*n* = 230)	OR	95%CI	*p*	Adj. OR	95%CI	*p*
Age (years)	32.9 ± 5.2	32.1 ± 5.2	1.032	0.969–1.098	0.326	-	-	-
BMI (kg/m^2^)	24.1 ± 4.1	22.8 ± 4.4	1.063	0.982–1.151	0.133	-	-	-
Secondary infertility	10 (22.1)	49 (21.3)	1.055	0.488–2.280	0.891	-	-	*-*
Incidental endometriosis	4 (8.9)	37 (16.1)	0.509	0.172–1.506	0.222	-	-	-
rASRM stage	2.4 ± 1.1	2.1 ± 1.1	1.400	1.018–1.926	*0.038*	1.400	1.018–1.926	*0.038*
Presence of an endometrioma	17 (37.8)	115 (50.0)	0.607	0.315–1.170	0.136	-	-	-
Presence of deep infiltrating endometriosis	5 (11.1)	24 (10.4)	1.073	0.386–2.979	0.893	-	-	-
Presence of peritubal adhesions	4 (8.9)	9 (3.9)	2.396	0.704–8.147	0.162			

**Table 5:** Prediction of uni- or bilateral fallopian tube occlusion in women with endometriosis using a binary logistic regression model.

	One or Both Tubes Closed	Bilateral Patency	Univariate Analysis			Multivariate Analysis		
	(*n* = 97)	(*n* = 178)	OR	95%CI	*p*	Adj. OR	95%CI	*p*
Age (years)	32.6 ± 5.5	32.0 ± 5.1	1.021	0.974–1.071	0.390	-	-	-
BMI (kg/m^2^)	23.8 ± 4.7	22.6 ± 4.2	1.063	0.995–1.135	0.071	-	-	-
Secondary infertility	29 (29.9)	30 (16.9)	2.104	1.171–3.779	*0.013*	2.181	1.191–3.995	*0.012*
Incidental endometriosis	10 (10.3)	31 (17.4)	0.545	0.255–1.166	0.118	-	-	-
rASRM stage	2.5 ± 1.0	2.0 ± 1.0	1.609	1.248–2.076	*<0.001*	1.514	1.156–1.983	*0.003*
Presence of an endometrioma	51 (52.6)	81 (45.5)	1.328	0.809–2.180	0.263	-	-	-
Presence of deep infiltrating endometriosis	17 (17.5)	12 (6.7)	2.940	1.340–6.449	*0.007*	2.045	0.888–4.713	0.093

**Table 6:** Results of chromopertubation according to the rASRM stage in the endometriosis group.

	rASRM I (*n =* 97)	rASRM II (*n =* 63)	rASRM III (*n =* 90)	rASRM IV (*n =* 25)	*p*
Unilateral tubal occlusion	12 (12.4)	9 (14.3)	25 (27.8)	6 (24.0)	*0.012*
Bilateral tubal occlusion	12 (12.4)	8 (12.7)	18 (20.0)	7 (28.0)	*0.043*
One or both tubes closed	24 (24.7)	17 (27.0)	43 (47.8)	13 (52.0)	*<0.001*

## Data Availability

The data are available from the corresponding author upon reasonable request.
